# Identification of factors related to functional prognoses in craniopharyngiomas

**DOI:** 10.1007/s11060-024-04925-7

**Published:** 2025-01-22

**Authors:** Tsuyoshi Umeda, Yoshihiro Otani, Kentaro Fujii, Joji Ishida, Shuichiro Hirano, Yasuki Suruga, Naoya Kemmotsu, Ryoji Imoto, Yasuhito Kegoya, Ryo Mizuta, Yohei Inoue, Madoka Hokama, Seiichiro Makihara, Kosei Hasegawa, Kenichi Inagaki, Fumio Otsuka, Takao Yasuhara, Shota Tanaka

**Affiliations:** 1 Department of Neurological Surgery, Okayama University Graduate School of Medicine, Dentistry and Pharmaceutical Sciences, 2-5-1 Shikata-cho, Kita-ku, Okayama, 700-8558 Japan; 2 Department of Otolaryngology-Head and Neck Surgery, Okayama University Graduate School of Medicine, Dentistry and Pharmaceutical Sciences, 2-5-1 Shikata-cho, Kita-ku, Okayama, 700-8558 Japan; 3 Department of Pediatrics, Okayama University Hospital, 2-5-1 Shikata-cho, Kita-ku, Okayama, 700-8558 Japan; 4 Department of Nephrology, Rheumatology, Endocrinology and Metabolism, Okayama University Graduate School of Medicine, Dentistry and Pharmaceutical Sciences, 2-5-1 Shikata-cho, Kita-ku, Okayama, 700-8558 Japan; 5 Department of General Medicine, Okayama University Graduate School of Medicine, Dentistry and Pharmaceutical Sciences, 2-5-1 Shikata-cho, Kita-ku, Okayama, 700-8558 Japan

**Keywords:** Craniopharyngioma, Functional prognosis, Obesity, Tumor size, Social participation, Hypothalamic involvement

## Abstract

**Purpose:**

Craniopharyngiomas are histologically benign tumors, but their proximity to vital neurovascular structures can significantly deteriorate functional prognoses and severely restrict patients’ social interaction and activity. We retrospectively identified risk factors related to the functional prognoses in patients with craniopharyngioma treated at our center.

**Methods:**

A retrospective analysis was conducted on 40 patients who underwent surgery for craniopharyngioma and follow-up at our institution between 2003 and 2022. Functional prognoses were evaluated in terms of obesity (body mass index [BMI] ≥ 25 for adults, BMI-Z ≥ 1.65 for children), visual function, endocrine function, and social participation. We investigated whether patient characteristics, tumor size, tumor location, hypothalamic involvement, surgical hypothalamic damage, extent of resection, and recurrence rate correlated with these functional prognostic factors.

**Results:**

The median age at diagnosis was 28.0 years, with a median follow-up of 80.5 months. Postoperative obesity was present in 22 patients, and those with postoperative obesity had a significantly higher preoperative BMI or BMI-Z (preoperative BMI for adults: *p* = 0.074; preoperative BMI-Z for children: *p* = 0.020) and were significantly correlated with preoperative hypothalamic involvement grade 2 (*p* = 0.012) and surgical hypothalamic damage grade II (*p* = 0.0001). Deterioration in social participation was significantly associated with a larger tumor size (*p* = 0.023) and tumor recurrence (*p* = 0.0047).

**Conclusions:**

Patients with higher preoperative BMI or BMI-Z and hypothalamic involvement have a greater risk of postoperative obesity, and larger tumor size and recurrence can significantly deteriorate the rate of patients’ social participation.

**Supplementary Information:**

The online version contains supplementary material available at 10.1007/s11060-024-04925-7.

## Introduction

Craniopharyngiomas (CPAs) are benign brain tumors arising from remnants of Rathke’s pouch. With separate prevalence peaks in childhood and adulthood, CPAs account for 2 to 5% of all primary intracranial neoplasms [1, 2]. Although histologically benign, their proximity to vital neurovascular structures, including the hypothalamus, pituitary gland, and visual pathway, can lead to a severe course of the disease characterized by memory and cognitive dysfunction, endocrinological deficiencies, visual deficits, and obesity, both at presentation and as a result of treatment [3–6]. With maximal surgical resection, overall survival rates are reported to be 83 to 90% at 10 years [7, 8]. Despite the high overall survival, the quality of life (QOL) for CPA patients might be significantly diminished by these comorbidities.

Historically, transcranial microsurgery (TCM) with gross-total resection (GTR) was the standard treatment for CPAs. However, the morbidities and mortality associated with this surgical treatment have made the management of CPAs challenging. Over the last decade, endoscopic endonasal surgery (EES) has been widely used for both sellar and suprasellar lesions [9, 10]. Combined with radiotherapy, long-term tumor control has also improved. Several reports suggest that radiosurgery is associated with 5-year tumor control rates of 62.2 to 73.6% [4, 11, 12].

While numerous reports exist on long-term tumor control and complication rates for surgical procedures, either alone or in combination with radiation therapy, there are limited reports that thoroughly explore the correlation between individual functional prognoses, such as endocrine function, visual function, obesity and return-to-work rates, and the factors influencing these prognoses. Yano et al. reported on the QOL of pediatric patients after long-term follow-up who underwent resection of CPAs [13]. In their report, long-term survivors lived independently but had a lower overall QOL. They emphasized the importance of preserving visual and hypothalamic function to enhance patients’ long-term QOL. Other studies indicated that higher preoperative BMI and hypothalamic involvement correlated with postoperative hypothalamic obesity [1, 5]. In this study, we conducted a retrospective investigation to identify the risk factors related to the individual functional prognoses after CPA treatment at our center.

## Methods

### Study design

In this retrospective study, patients with CPA who underwent TCM or EES at Okayama University Hospital (OUH) between December 2003 and November 2022 were included. Patients were excluded if they only underwent biopsy surgery or were treated conservatively. This study received approval from the institutional review board at OUH (1608-026).

### Data collection

Electronic medical records were reviewed, including baseline characteristics (age at initial surgery, gender, preoperative BMI for adults or BMI Z score (BMI-Z) for children, visual status, endocrinological status, tumor location, hypothalamic involvement, tumor size), surgical approach, radiotherapy, pathological diagnosis, and postoperative outcomes (extent of resection (EOR), surgical hypothalamic damage, recurrence, progression-free survival, overall survival, postoperative BMI for adults or BMI-Z for children, visual status, endocrinological status, return to work or school). BMI was calculated as body weight (kg) divided by height (m) squared (kg/m^2^), using weight and height at the time of diagnosis and final follow-up. BMI-Z was calculated using the formula (BMI for each patient / M)^L^ / (L * S), where L, M, and S represent the Box-Cox power parameter, the median BMI for each patient’s age and gender, and the coefficient of variation parameter, respectively. Obesity was defined by either BMI ≥ 25 or BMI-Z ≥ 1.65 [5].

Pituitary gland dysfunction was defined as the need for hormone replacement. Anterior pituitary gland dysfunction was defined as the requirement for any hormonal replacement therapy. Posterior pituitary gland dysfunction was determined by the presence of arginine vasopressin deficiency. Partial hypopituitarism was defined as the need for one or two hormone replacements, while panhypopituitarism was defined as the need for three or more hormone replacements [14]. Visual dysfunction was defined as any changes in vision (visual fields or visual acuity). Visual function tests were conducted in all patients both before and after the surgery, except for those who were too young to conduct the examinations (*n* = 2).

Social participation was evaluated separately for children, adults, and the elderly at the last outpatient follow up. Children were evaluated based on school attendance and participation in regular classes, adult were assessed on social reintegration, and the elderly were evaluated for their independence in ADLs using the modified Rankin Scale ranging from 0 to 2. Social reintegration was assessed based on whether the individual returned to work in a job with a guaranteed minimum wage, or, for homemakers, whether they were able to manage household chores. Changes in job responsibilities or occupation were also considered acceptable. Tumor location, hypothalamic involvement, and tumor size were determined using preoperative magnetic resonance imaging (MRI) or computed tomography. Tumor location was categorized into two groups: sellar-suprasellar or sellar-suprasellar and hypothalamus regions. Hypothalamic involvement was classified into three grades as follows: grade 0, tumor at a distance from the hypothalamus, corresponding to the “sellar-suprasellar” tumor location; grade1, tumor in contact with the hypothalamus with negligible hypothalamic damage; grade 2, tumor spread to the hypothalamus, which is no longer identifiable. Grades 1 and 2 correspond to the “sellar-suprasellar and hypothalamus” tumor location [15, 16]. Tumor size was measured as the maximum diameter (mm). EOR was classified as either GTR or subtotal resection (STR). GTR was defined as resection with no residual tumor confirmed on postoperative MRI obtained within 48 h after surgery. If any evidence of residual tumor was present on postoperative MRI, the EOR was classified as STR. Surgical hypothalamic damage was classified into three grades based on MRI findings within six months following surgery: grade 0, no hypothalamic damage; grade I, negligible hypothalamic damage or residual tumor displacing the hypothalamus; grade II, significant hypothalamic damage (floor of the third ventricle not identifiable) [17]. Recurrence was defined as the emergence of tumors detected on neuroimaging for patients who underwent GTR or the progression of a residual tumor. Progression-free survival (PFS) was defined as the period from surgery to the occurrence of recurrence.

### Statistical analysis

Statistical analysis was conducted using GraphPad Prism 10 (La Jolla, CA, USA). Continuous variables were described using the mean ± standard deviation or the median (whole range) as appropriate. The Mann–Whitney U test or Student’s *t*-test was used to compare continuous variables between two independent groups. The Chi-square test or Fisher’s exact test was used to compare categorical variables. PFS and overall survival (OS) were estimated using Kaplan–Meier analyses. Two-sided *p*-values less than 0.05 were considered statistically significant.

## Results

### Patient characteristics

A total of 40 patients with CPA were identified, of whom 60.0% were male, with a median age at initial surgery of 28.0 years (range: 0–75 years). The median follow-up period was 80.5 months (range: 3–229 months) (Table 1). The mean preoperative BMI for adults was 25.1 **±** 3.9 (range: 19.9–35.0), and the mean preoperative BMI-Z for children was 0.43 **±** 0.96 (range: 1.0–2.0). Tumors located in the sellar-suprasellar accounted for 35.0% of cases, while 65.0% were in the sellar-suprasellar and hypothalamus regions. As for hypothalamic involvement, grades 0, 1 and 2 accounted for 35.0%, 20.0% and 45.0% respectively. The median tumor size was 31.8 mm (range: 12.3–90.0 mm). A total of 30 (75.0%) patients had preoperative visual impairment. For the initial treatment, 17 (42.5%) patients underwent EES, and 22 patients underwent TCM. One patient (2.5%) underwent TCM and EES simultaneously. Twelve (30.0%) patients underwent radiation therapy: 3 (25.0%) of them received radiation therapy for residual disease following the initial surgery, and 9 (75.0%) received it at the time of recurrence. The histopathological diagnosis was as follows: 34 (85.0%) were adamantinomatous type, and 6 (15%) were papillary type.

**Table 1:** Patient characteristics and clinical outcomes (*n* = 40)

Patient Characteristics	
Median follow-up, months (range)	80.5 (3–229)
Median patient age, years (range) Children (0–18 years old), n (%) Adults (18–65 years old), n (%) Elderly (65 + years old), n (%)	28.0 (0–75) 14 (35.0%) 21 (52.5%) 5 (12.5%)
Gender	
Male, n (%) Female, n (%)	24 (60.0%) 16 (40.0%)
Preoperative BMI, mean ± SD (range), for adults Preoperative BMI-Z, mean ± SD (range), for children	25.1 **±** 3.9 (19.9–35.0) 0.43 **±** 0.96 (-1.0–2.0)
Preoperative visual impairment, n (%)	30 (75.0%)
Median tumor size, mm (range)	31.8 (12.3–90.0)
Tumor location	
Sellar-suprasellar, n (%) Sellar-suprasellar + Hypothalamus, n (%)	14 (35.0%) 26 (65.0%)
Hypothalamic involvement	
Grade 0, n (%) Grade 1, n (%) Grade 2, n (%)	14 (35.0%) 8 (20.0%) 18 (45.0%)
Initial surgical approach	
TCM, n (%) EES, n (%) TCM + EES, n (%)	22 (55.0%) 17 (42.5%) 1 (2.5%)
Radiation therapy	
None, n (%) Post initial surgery, n (%) At the time of recurrence, n (%)	28 (70.0%) 3 (7.5%) 9 (22.5%)
Histopathological diagnosis	
Adamantinomatous type, n (%) Papillary type, n (%)	34 (85.0%) 6 (15.0%)
Clinical Outcomes	
Extent of resection	
GTR, n (%) STR, n (%)	17 (42.5%) 23 (57.5%)
Surgical hypothalamic damage	
Grade 0, n (%) Grade I, n (%) Grade II, n (%)	12 (30.0%) 10 (25.0%) 18 (45.0%)
Recurrence, n (%)	22 (55.0%)
Progression-free survival, months (range)	21.5 (1–155)
Postoperative BMI, mean ± SD (range), for adults Preoperative BMI-Z, mean ± SD (range), for children	25.2 **±** 3.4 (17.5–30.8) 0.63 **±** 1.2 (-1.7–2.6)
Postoperative visual function Improved, n (%) No change, n (%) Deterioration, n (%)	30 (75.0%) 13 (43.3%) 15 (50.0%) 2 (6.7%)
Postoperative endocrinological status	
Anterior pituitary gland dysfunction, n (%) Adrenocorticotropic hormone replacement, n (%) Thyroid hormone replacement, n (%) Growth hormone replacement, n (%) Gonadotropic hormone replacement, n (%) Posterior pituitary gland dysfunction, n (%)	37 (92.5%) 37 (92.5%) 34 (85.0%) 11 (27.5%) 12 (30%) 32 (80.0%)
Social participation	
Children– attendance of regular classes (0–18 years old), n (%) Adults– social reintegration (18–65 years old), n (%) The elderly– ADL independence (65 + years old), n (%)	11 (78.6%) 19 (90.5%) 2 (40.0%)

TCM = transcranial microsurgery, EES = endoscopic endonasal surgery. GTR = gross-total resection, STR = subtotal resection

### Clinical outcomes

GTR was achieved in 42.5% (17/40) of patients, while STR was performed in 57.5% (23/40) (Table 1). Regarding surgical hypothalamic damage, grades 0, I and II accounted for 30.0%, 25.0% and 45.0% respectively. During the follow-up period, recurrence occurred in 55.0% (22/40) of patients, and the median PFS was 21.5 months (range: 1–155 months). The mean postoperative BMI for adults was 25.2 **±** 3.4 (range: 17.5–30.8), and the mean postoperative BMI-Z for children was 0.63 **±** 1.2 (range: 1.7–2.6), indicating a slight increase from the preoperative BMI. Among the 30 patients with preoperative visual impairment, 43.4% (13/30) showed improvement after surgery, 50% (15/30) experienced no significant change, and 6.7% (2/30) exhibited deterioration. Postoperatively, 92.5% (37/40) of patients required some form of permanent hormone replacement for anterior pituitary gland dysfunction. Permanent arginine vasopressin deficiency, resulting from dysfunction of the posterior pituitary gland, was present in 80% (32/40) of patients. Concerning social participation, 78.6% (11/14) of the pediatric patients were able to attend school and regular classes, and 90.5% (19/21) of the adults achieved social reintegration. In contrast, only 40% (2/5) of the elderly achieved　independence in ADLs.

### Univariate analysis for functional prognoses

**Fig. 1:**
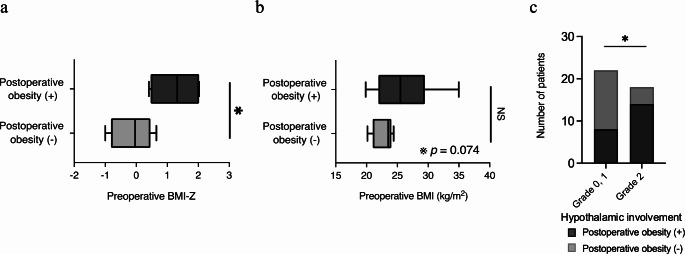
Comparison of pre and postoperative obesity. Boxplots illustrating the mean and distribution of preoperative BMI for adults with postoperative obesity compared to those with postoperative non-obesity (**a**), and the mean and distribution of preoperative BMI-Z for children with postoperative obesity compared to those with postoperative non-obesity (**b**). Number of patients with postoperative obesity and non-obesity stratified by hypothalamic involvement (**c**)

Univariate analysis was conducted to identify functional prognostic factors related to postoperative obesity, endocrine function, visual function, and social participation. The analysis revealed that a higher preoperative BMI-Z for children significantly correlated with postoperative obesity (preoperative BMI-Z for children: 1.3 vs. -0.13, *p* = 0.020), and there was a trend towards correlation between higher preoperative BMI for adults and postoperative obesity (preoperative BMI for adults: 26.1 kg/m^2^ vs. 22.8 kg/m^2^, *p* = 0.074) (Fig. 1a and b; Table 2). Furthermore, hypothalamic involvement grade 2 was significantly associated with postoperative obesity compared to hypothalamic involvement grades 0 and 1 (*p* = 0.012) (Fig. 1c). Surgical hypothalamic damage grade II was also associated with postoperative obesity (*p* = 0.0001).

**Table 2:** Univariate analysis for postoperative obesity

Variables	Obesity (*n* = 22)	Non-obesity (*n* = 18)	*p* value
Age at initial operation (years)			0.64
< 18 ≥ 18	7 15	7 11	
Gender			> 0.99
Male Female	13 9	11 7	
Extent of resection			> 0.99
GTR STR	9 13	8 10	
Radiation therapy			0.32
Yes No	5 17	7 11	
Recurrence			> 0.99
Yes No	12 10	10 8	
Tumor location			0.33
Sellar-suprasellar Sellar-suprasellar + Hypothalamus	6 16	8 10	
Hypothalamic involvement			**0.012**
Grade 0, 1, n (%) Grade 2, n (%)	8 14	14 4	
Surgical hypothalamic damage			**0.0001**
Grade 0, I, n (%) Grade II, n (%)	6 16	16 2	
Tumor size– maximum diameter, mm (range)	31.6 (12.3–69.3)	32.5 (16.5–90.0)	0.86
Anterior pituitary gland dysfunction			0.58
Yes No	21 1	16 2	
Posterior pituitary gland dysfunction			0.71
Yes No	17 5	15 3	
Preoperative BMI, mean ± SD (range), for adults Preoperative BMI-Z, mean ± SD (range), for children	26.1 **±** 4.3 (19.9–35.0) 1.3 **±** 0.75 (0.63–2.03)	22.8 **±** 3.7 (20.1–24.4) -0.13 **±** 0.61 (-1.0–0.65)	0.074 **0.020**

**Fig. 2:**
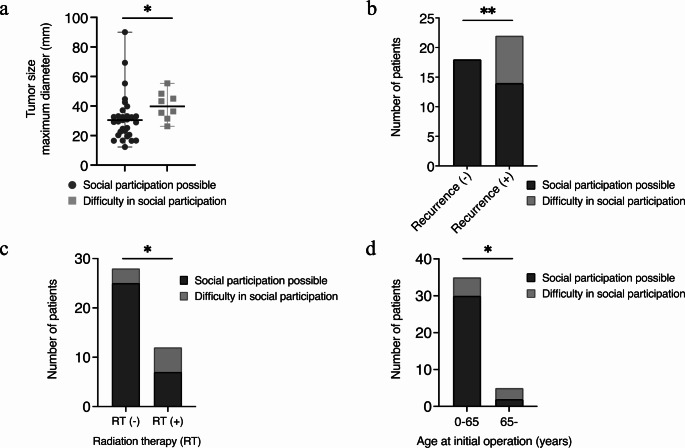
Factors related to social participation. Scatter plot illustrating the median tumor size for individuals with possible social participation compared to those experiencing challenges in social participation (**a**). Number of patients with possible social participation and challenges in social participation stratified by recurrence status (**b**), radiation therapy history (**c**), and age at initial operation (**d**)

Regarding social participation, larger tumor size (39.8 mm vs. 30.4 mm, *p* = 0.023), recurrence (*p* = 0.0047), and radiation therapy (*p* = 0.039) were significantly associated with poor prognosis (Fig. 2a, b and c; Table 3). In addition, while age at the initial operation (< 18 or ≥ 18) did not exhibit a significant relationship with social participation, a notable difference was observed when age was categorized into < 65 years and ≥ 65 years. Social participation was significantly more challenging for individuals aged 65 years and older (*p* = 0.046) (Fig. 2d). No significant factors were identified related to endocrine or visual dysfunction (Supplementary Tables 1, 2, 3).

**Table 3:** Univariate and Multivariate analysis for social participation

Variables	Univariate			Multivariate	
	Possible (*n* = 32)	Difficult (*n* = 8)	*p* value	OR (95% confidence interval)	*p* value
Age at initial operation (years)			> 0.99	NA	NA
< 18 ≥ 18	11 21	3 5			
			**0.046**	14.64 (1.356–292.1)	**0.039**
< 65 ≥ 65	30 2	5 3			
Gender			0.69	NA	NA
Male Female	20 12	4 4			
Extent of resection			0.11	NA	NA
GTR STR	16 16	1 7			
Radiation therapy			**0.039**	14.64 (1.889–242.3)	**0.022**
Yes No	7 25	5 3			
Recurrence			**0.0047**	NA	NA
Yes No	14 18	8 0			
Tumor location			0.22	NA	NA
Sellar-suprasellar Sellar-suprasellar + Hypothalamus	13 19	1 7			
Hypothalamic involvement			0.11	NA	NA
Grade 0, 1, n (%) Grade 2, n (%)	20 12	2 6			
Surgical hypothalamic damage			0.11	NA	NA
Grade 0, I, n (%) Grade II, n (%)	20 12	2 6			
Tumor size– maximum diameter, mm (range)	30.4 (12.3–90.0)	39.8 (26.3–55.3)	**0.023**	1.063 (0.9995–1.146)	0.061
Anterior pituitary gland dysfunction			> 0.99	NA	NA
Yes No	29 3	8 0			
Posterior pituitary gland dysfunction			0.65	NA	NA
Yes No	26 6	6 2			
Preoperative BMI, mean ± SD (range), for adults Preoperative BMI-Z, mean ± SD (range), for children	25.2 **±** 4.3 (19.9–35.0) 0.43 **±** 1.0 (-1.0–2.03)	24.5 **±** 3.5 (21.4–28.2) 0.39 **±** 0 (0.39)	0.74 0.97	NA NA	NA NA

The variable “Recurrence” was excluded because of perfect separation error in the statistical calculation

### Multivariate analysis for functional prognoses

Regarding social participation, multivariate analysis was conducted on variables that were significantly different in the univariate analysis, excluding recurrence due to impossibility of statistical calculation caused by perfect separation error. The analysis revealed that age at the initial operation (≥ 65 years) (*p* = 0.039) and radiation therapy (*p* = 0.022) were significantly correlated with difficulty in social participation. Additionally, a larger tumor size showed a significant trend toward poor prognosis (*p* = 0.061) (Table 3).

## Discussion

Although CPA is typically considered a benign brain tumor, its clinical impact can be challenging due to its location and potential for aggressive behavior. In this retrospective analysis, we comprehensively examined clinical factors affecting patient outcomes, focusing on obesity, visual function, endocrine function, and social impairment. Our findings underscored the association between preoperative obesity and the risk of postoperative obesity, particularly among children. Preoperative hypothalamic involvement was also found to have a significant correlation with postoperative obesity. Surgical hypothalamic damage was associated with postoperative obesity, which is likely due to the strong correlation between surgical hypothalamic damage and preoperative hypothalamic involvement. Patients with larger tumors and those who received radiation therapy exhibited poorer social participation outcomes. Elderly patients, in particular, faced challenges in social prognosis.

Postoperative obesity represents a significant complication of CPA, contributing to increased risks of metabolic and cardiovascular diseases, diminishing QOL, and excess mortality [1, 18, 19]. Wei et al. conducted a single-institution analysis on the risk factors for postoperative hypothalamic obesity in patients with adult-onset CPA. The study revealed that the prevalence of preoperative hypothalamic obesity increased postoperatively from 19.2 to 29.2%. They identified that preoperative BMI was an independent risk factor for postoperative hypothalamic obesity. Müller et al. investigated the risk factors for obesity in patients with childhood CPA and found a significant correlation between preoperative hypothalamic involvement and obesity [20–22]. In their study, high preoperative BMI (BMI ≥ 25) was also significantly associated with postoperative obesity. These findings were consistent with our results.

As mentioned above, hypothalamic involvement is closely associated with obesity. However, there are also reports suggesting that endocrine and metabolic abnormalities contribute to obesity. Iughetti et al. reported that while hypothalamic involvement is a primary factor in causing obesity, other pituitary hormone deficiencies are also associated with this condition [23]. For example, growth hormone deficiency can result in an increased percentage of body fat, abnormal lipoprotein metabolism, and increased peripheral insulin resistance, leading to impaired glucose tolerance [24, 25]. In addition, hypothyroidism and hypocortisolism decrease energy metabolism and physical activity. Thus, these hormone deficiencies also lead to obesity.

There are limited reports investigating social participation among postoperative CPA patients. In our study, we observed significant challenges in social participation among patients with tumor recurrence and those with larger tumor sizes. To the best of our knowledge, there are few reports on how tumor size or recurrence impacts social participation. Emphasizing maximal safe resection while minimizing treatment complications, along with the use of and BRAF-MEK inhibitors mentioned later, could enhance long-term tumor control and thereby improve social participation outcomes. However, in pediatric craniopharyngioma patients, it should be noted that although they appear to have normal cognitive and mental functions, they may still face varying degrees of difficulties in daily life, such as interpersonal relationships, emotional control, and learning [26, 27]. In our report, 78.6% (11/14) of the pediatric patients have advanced to regular class, but even among them, some may be withdrawn, struggle with studies, or be concerned about their appearance due to short height and obesity. Our finding also highlights that patients aged 65 years and older face significant challenges in achieving independence in ADLs. For elderly patients, opting for less invasive surgeries and preventing surgery-related complications, or limiting the procedure to STR with subsequent radiation therapy, might be prudent [28]. However there remains a paucity of research on CPA in elderly populations, and further investigation is needed. The relationship between radiation therapy (*p* = 0.039) and social participation should be interpreted carefully, given that it was predominantly administered in cases of recurrence in this study. Recurrence was significantly correlated with challenges in social participation, implying a strong selection bias regarding radiation therapy.

Although medications historically had limited effectiveness for CPAs, recent reports highlight a significant impact of BRAF-MEK inhibitors on papillary CPAs. BRAF V600E mutations are present in over 90% of papillary CPA cases, and these inhibitors have shown considerable efficacy in both initial treatments and recurrent cases [29–32]. The adverse events associated with these drugs have generally been manageable. Despite the lower incidence of papillary CPAs compared to adamantinomatous CPAs, the use of BRAF-MEK inhibitors instead of radical surgery may improve patient outcomes.

The limitations of this study include its retrospective design conducted at a single institution and the relatively small number of cases. In addition, the long study period from 2003 to 2022 encompasses advancements in surgical methods and medications, potentially influencing the functional prognoses assessed.

## Conclusions

We retrospectively identified risk factors influencing functional outcomes following CPA treatment at our center. Higher preoperative BMI or BMI-Z, hypothalamic involvement and surgical hypothalamic damage were associated with postoperative obesity, whereas larger tumor size, recurrence, and age at the initial operation (≥ 65 years) were significantly associated with a poorer social participation prognosis. Further investigation into risk factors affecting functional outcomes requires a larger sample size.

## Electronic supplementary material

Below is the link to the electronic supplementary material.

Supplementary Material 1

## Data Availability

No datasets were generated or analysed during the current study.
