# Supplementary material for: Identification of factors related to functional prognoses in craniopharyngiomas
Source: J Neurooncol. 2025 Jan 22;172(2):471–9. doi: 10.1007/s11060-024-04925-7 (PMC11937222; doi:10.1007/s11060-024-04925-7)
Supplement: Supplementary file 1 — Supplementary Material 1 [file 11060_2024_4925_MOESM1_ESM.doc]

Supplementary Table 1. Univariate analysis for postoperative anterior pituitary gland dysfunction (hormone replacement)

| Variables | Yes (n = 37) | No (n = 3) | *p* value |
| --- | --- | --- | --- |
| Age at initial operation (years)  < 18  ≥ 18 | 14  23 | 0  3 | 0.54 |
| Gender  Male  Female | 23  14 | 1  2 | 0.55 |
| Extent of resection  GTR  STR | 16  21 | 1  2 | > 0.99 |
| Radiation therapy  Yes  No | 12  25 | 0  3 | 0.54 |
| Recurrence  Yes  No | 16  21 | 1  2 | 0.58 |
| Tumor location  Sellar-suprasellar  Sellar-suprasellar + Hypothalamus | 12  25 | 2  1 | 0.28 |
| Hypothalamic involvement  Grade 0, 1, n (%)  Grade 2, n (%) | 20  17 | 2  1 | > 0.99 |
| Surgical hypothalamic damage  Grade 0, I, n (%)  Grade II, n (%) | 20  17 | 2  1 | > 0.99 |
| Tumor size – maximum diameter, mm (range) | 32.5 (12.3–90.0) | 20.5 (16.5–29.2) | 0.078 |
| Posterior pituitary gland dysfunction  Yes  No | 30  7 | 2  1 | 0.50 |
| Preoperative BMI, mean ± SD (range), for adults  Preoperative BMI-Z, mean ± SD (range), for children | 24.6 **±** 3.4 (19.9–33.3)  0.43 **±** 0.96 (-1.00–2.03) | 29.2 **±** 5.7 (23.5–35.0)  NA | 0.13  NA |

GTR = gross-total resection, STR = subtotal resection

Supplementary Table 2. Univariate analysis for postoperative posterior pituitary gland dysfunction (hormone replacement)

| Variables | Yes (n = 32) | No (n = 8) | *p* value |
| --- | --- | --- | --- |
| Age at initial operation (years)  < 18  ≥ 18 | 13  19 | 1  7 | 0.22 |
| Gender  Male  Female | 19  13 | 5  3 | > 0.99 |
| Extent of resection  GTR  STR | 15  17 | 2  6 | 0.43 |
| Radiation therapy  Yes  No | 9  23 | 3  5 | 0.68 |
| Recurrence  Yes  No | 18  14 | 4  4 | > 0.99 |
| Tumor location  Sellar-suprasellar  Sellar-suprasellar + Hypothalamus | 22  10 | 5  3 | > 0.99 |
| Hypothalamic involvement  Grade 0, 1, n (%)  Grade 2, n (%) | 17  15 | 5  3 | 0.709 |
| Surgical hypothalamic damage  Grade 0, I, n (%)  Grade II, n (%) | 17  15 | 5  3 | 0.709 |
| Tumor size – maximum diameter, mm (range) | 32.6 (12.3–90.0) | 26.9 (16.7–69.3) | 0.43 |
| Anterior pituitary gland dysfunction  Yes  No | 30  2 | 7  1 | 0.50 |
| Preoperative BMI, mean ± SD (range), for adults  Preoperative BMI-Z, mean ± SD (range), for children | 24.2 **±** 2.6 (19.9–29.5)  0.43 **±** 1.0 (-1.00–2.03) | 27.4 **±** 5.6 (21.0–35.0)  0.39 **±** 0 (0.39) | 0.098  0.97 |

Supplementary Table 3. Univariate analysis for postoperative visual function

| Variables | Improved (n = 13) | No change /  deterioration (n = 17) | | *p* value |
| --- | --- | --- | --- | --- |
| Age at initial operation (years)  < 18  ≥ 18 | 4  9 | | 6  11 | > 0.99 |
| Gender  Male  Female | 10  3 | | 8  9 | 0.14 |
| Initial surgical approach  TCM  EES  TCM + EES | 6  6  1 | | 12  5  0 | 0.33 |
| Extent of resection  GTR  STR | 6  7 | | 6  11 | 0.71 |
| Radiation therapy  Yes  No | 5  8 | | 5  12 | 0.71 |
| Recurrence  Yes  No | 5  8 | | 11  6 | 0.27 |
| Tumor location  Sellar-suprasellar  Sellar-suprasellar + Hypothalamus | 5  8 | | 5  12 | 0.71 |
| Hypothalamic involvement  Grade 0, 1, n (%)  Grade 2, n (%) | 8  5 | | 9  8 | 0.72 |
| Surgical hypothalamic damage  Grade 0, I, n (%)  Grade II, n (%) | 8  5 | | 9  8 | 0.72 |
| Tumor size – maximum diameter, mm (range) | 31.4 (12.3–36.4) | | 33.1 (16.6–90.0) | 0.21 |
| Anterior pituitary gland dysfunction  Yes  No | 12  1 | | 16  1 | > 0.99 |
| Posterior pituitary gland dysfunction  Yes  No | 12  1 | | 11  6 | 0.10 |
| Preoperative BMI, mean ± SD (range), for adults  Preoperative BMI-Z, mean ± SD (range), for children | 24.3 **±** 2.8 (21.4–29.5)  0.33 **±** 1.0 (-1.00–2.03) | | 25.6 **±** 4.6 (21.0–35.0)  -0.023 **±** 0 (-0.75–0.39) | 0.53  0.69 |
